# Impact of the immobilized *Bacillus cereus* MG708176 on the characteristics of the bio-based self-healing concrete

**DOI:** 10.1038/s41598-023-27640-1

**Published:** 2023-01-10

**Authors:** Amany M. Reyad, Gehad Mokhtar

**Affiliations:** 1 Department of Botany, Faculty of Science, Fayoum University, Faiyum, Egypt; 2 Civil Engineering, Future High Institute of Engineering in Fayoum, Faiyum, Egypt

**Keywords:** Microbiology, Engineering

## Abstract

Novel carrier units were evaluated for their bio-healing benefits in our study to increase the efficacy of concrete healing. *Bacillus cereus* MG708176, an alkali-tolerant, calcite precipitating, endospore-forming strain was added as a bio-healing agent after its immobilization on wood ash units. A spore concentration of [1.3 × 10^7^ spore/cm^3^] combined with 2.5% w/w urea was added to cement. Beams of 40 × 40 × 160 mm were used and tested for completely damaged mortar specimens after 7, 14, and 28 days of water treatment. Using wood ash bacterial mortars, totally destructed specimens were fully healed in all time intervals. Positive changes in concrete mechanical properties in bacterial wood ash treatment that were 24.7, 18.9, and 28.6% force for compressive, flexural, and tensile strengths more than control. The micro-images of the Scanning Electron Microscope (SEM) showed the dense concrete structure via calcite, Bacillafilla, and ettringite formation. Our results have shown improvements in the concrete healing efficiency and the mechanical concrete properties by filling the concrete cracks using a calcite-producing bacterium that is immobilized on wood ash units.

## Introduction

Bio-healing is a process that is biologically developed by certain microorganisms to heal cracks in concrete structures suffering from cracking that causes deterioration and shorter service life^1,2^. Microbial CaCO_3_ is an ecofriendly and cost-effective material with a large range of engineering applications that are promising^3^. Bacteria have a natural capability to heal minor damage on their bodies in a relatively short time, without external influences^4^. The natural concrete ability of self-healing by the swelling of the cement particles due to un-hydrated particles hydration could occur without adding healing agents^5,6^.

The efficient ingredient of bacterial-induced calcite precipitation (BICP) is urea because urease is well-covered across bacteria. Urease activity leads to pH increase and generation of nitrogen, hydroxides, and bicarbonate^7^. The urease activity of BICP enables them for use in the fields of environmental studies^8^.

It is worth noting that during the hydration process for cement, the bacterial cells or spores may be exposed to a risk of damage^9^. Bacterial introduction into concrete without using carrier compounds dramatically reduces bacterial survival over time^10^. As a result, to improve the efficacy of the bio-healing process, researchers have used different carrier compounds to strengthen bacterial survival inside the concrete. It is beneficial to use these carrier compounds, not only for increasing bacterial survival chances but also increase the concrete tensile strength^11^.

An attempt to treat the bacterial survival problem is by using bacterial carriers as encapsulation and bacterial immobilization on different carriers. As cracks occur, under the crack-tip tension, the embedded spores in crack surfaces are drawn into the crack and germinate to achieve healing^12^. In our study, a new calcite forming bacterial isolate was inserted into the matrix of concrete and the impacts of the wood ash carrier on the bacterial capacity for bio-healing were investigated. These findings are used to compare a direct bacterial spore inoculation and the bacterial spores’ immobilization on wood ash to select the best technique for the introduction of spores into the concrete. According to a survey of the literature, wood ash is the burning wood waste (chips, extracts, sawdust, etc.) and the most important components in wood ash are CaO, SiO_2_, Al_2_O_3_, Fe_2_O_3_, and MgO, which react with moisture to form bonding agents^13,14^. Wood ash is a freely available agricultural waste that increases the quality, microstructure, use, and strength of concrete^15^. As a result, using wood ash in concrete is preferable because unburned carbon in wood ashes affects the level of pozzolanic material in the ash^14^. The use of wood ash units as a bacterial carrier is a promising method because, although it is thought of as a waste, it enhances the mechanical properties of produced composites, resulting in better strength and stiffness mixtures^16^ and offering inexpensive raw materials for the immobilization process. Using wood ash units improves concrete characteristics and that makes us to introduce a new approach to use it as a bacterial carrier to protect bacteria from harsh conditions of concrete.

## Materials and methods

### Bacterial characterization

An endospore-forming bacterium was isolated and identified as previously described by Hemida and Reyad^17^. The procedure of the endospore staining was performed as shown by Mokhtar et al*.*^18^. Alkali solid medium was prepared by adding NaOH solution droplets until pH = 14 to nutrient agar (NA) (5 g peptone, 3 g beef extract, 8 g sodium chloride, and 15 g agar dissolved 1000 mL distilled water) and the bacterial isolate was cultivated on its surface^18^. The urease test described by Christensen^19^ was made. CaCO_3_ precipitation test was made as described by Fujita et al*.*^20^.

### Bio-healing agent preparation

For the bacterial spores harvesting, the bacterial isolate was cultured for 24 h in alkaline nutrient broth (D (+)-glucose, 1 g/L, peptone, 15 g/L, sodium chloride, 6 g/L, yeast extract, 3 g/L, and then adding NaOH solution droplets until pH 14). The amended spore concentration in samples was 1.3 × 10^7^ spore/cm^3^ of the entire concrete mixture^18^. The nutrients, including 40 g/L calcium chloride anhydrous, 65 g/L urea, and 2 g/L yeast extract were dissolved in sterilized tap water for preparing the bio-based concrete.

Wood ash units of 0.2 mm particle size were created and impregnated with a mixture of nutrients and bacterial spores. Sawdust was obtained from carpentry shops and burned in an electric oven at 570° C. SEM–EDX analysis was used to characterize the wood ash (see supplementary file). Spores of a bacterial isolate obtained by culturing the bacteria in alkaline NB for 72 h. For the harvesting of the bacterial spores, multiple centrifugation steps in dual sterilized tap water were done for obtaining bacterial cultures of a high number of spores. Suspensions were heated at 80° C for 35 min to inactivate existing vegetative cells, and the usual count cultivation-dilution technique quantified the viable spores’ number in suspensions. Wood ash was sieved by a 0.2 mm sieve to get rid of the coarse particles in the ash before being used for the immobilization process. Wood ash units impregnated with nutrients and spores were dried in an oven for 5 days at 37 °C and ready to be inserted in the mixture of mortar or concrete.

### Experimental design

The testing program was conducted following Egyptian Code Practice (ECP)^21^, and American Society for Testing and Materials (ASTM) standards^22^. The study consisted of two consecutive steps. Firstly, designed three different mixture types (mortar specimens), and tested them under direct incorporation and one carrier method. The spore suspension was injected directly during the last stage of the concrete mixing process.
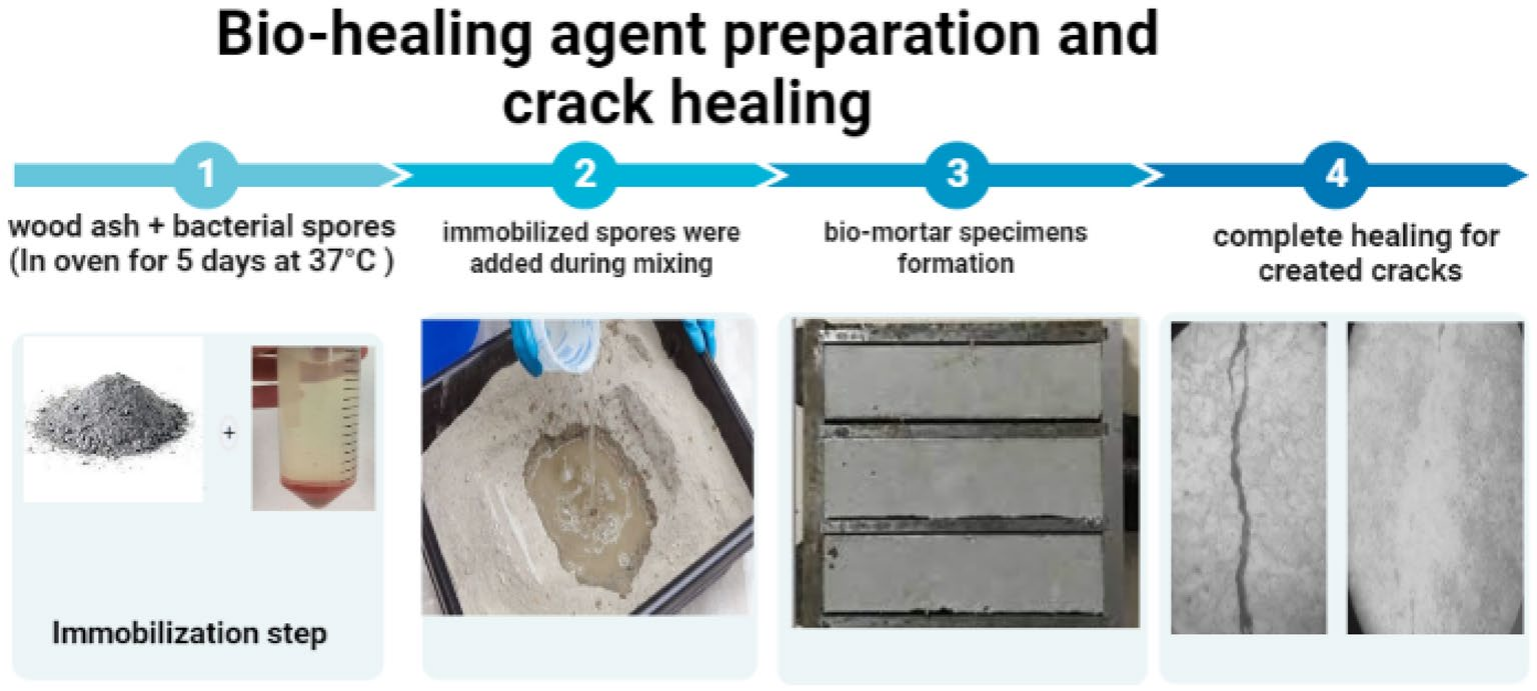


Subsequently, only the best technique (bacterial wood ash) was chosen for the introduction into a concrete mixture. Negative control samples of mortar were named “C1” in which no bacterial spores or wood ash components were added. Wood ash only without bacterial spores, termed as “C2” was considered a positive control. In ''T1” specimens, Bacteria have been directly inserted into concrete ingredients without using any protective carrier compounds. “T2” is designated for the integration with bacteria as defensive carriers through using the wood ash units. Table 1 shows the mixture proportions for the mortar and concrete mixtures composition. The mixture proportions for the concrete samples, control specimens of the concrete were named “C” in which there were no bacterial spores. Using the wood ash as the defensive carrier, bacteria were introduced and denoted as “T”.

**Table 1:** The mixture design of all specimens.

Mixture Type	Water (kg/m^3^)	Cement (kg/m^3^)	Sand (kg/m^3^)	Bacterial spores 1.3 × 10^7^ spore/cm^3^	Wood ash (kg/m^3^)	Aggregate (kg/m^3^)
C1	140	350	1050	− ve	–	–
C2	140	350	1047	− ve	3	–
T1	140	350	1050	+ ve	–	–
T2	140	350	1047	+ ve	3	–
C	150.8	350	673.5	− ve	3	1340.2
T	150.8	350	673.5	+ ve	3	1340.2

C1: Negative control (all mortar constituents without wood ash or bacterial spores). C2: Positive control (all mortar constituents + wood ash). T1: All mortar constituents + direct bacterial spores inoculation. T2: All mortar constituents + immobilized bacterial spores on wood ash. C: all concrete constituents + wood ash. T: all concrete constituents + wood ash + bacterial spores.

### Test specimens

The removal of the specimens was taken place after 24 h casting and then cured with water. Standard molds of Beam for mortar specimens (40 × 40 × 160 mm) were used. Compression and self-healing were conducted at various curing times of 7, 14, and 28 days for hardened concrete. The compressive, flexural, and indirect tests of splitting tensile strength for hardened concrete after 28 days were conducted for concrete specimens. Cylinder molds of 100 × 200 mm, 100 mm cubic, 100 × 100 × 500 mm beam were used. Besides, to track microstructural changes due to calcite formation, samples were also subjected to a SEM. Bacterial calcite precipitation was scanned by using SEM micrographs in micro-cracks specimens. These micrographs were created using a Carl Zeiss sigma 500 VP. A stereomicroscope was used for mortar self-healing measurements.

### Concrete slump analysis

For measuring the strength of fresh concrete, the slump test is widely used. It was achieved per ASTM C143^23^. For the slump test, a cone-shaped metallic mold with internal dimensions of a 20 cm bottom diameter, a top diameter of 10 cm, and 30 cm in height. This mold is settled on a surface that is flat, horizontal, and non-absorbent. A fresh concrete sample test must be taken immediately after mixing with a pan blender and put in a three-layer cone mold; a 25 times compaction for each one of the layers was taken place by a standard tamping rod. The mold of the cone is automatically separated from the concrete by moving it slowly and carefully in a vertical direction. This allows the concrete to recede. The mean level between the height of the mold and the highest point of the subsided concrete shall be measured.

### Compressive strength analysis (σ)

After 28th days from casting, 100 × 100 mm cube specimens were subjected to ECP 203 compression testing. To conduct the test, a two thousand KN (ADR 2000) compression testing system was used. Specimens were positioned following the ISO 4012 standard requirements on a rigid bottom bearing block with a spherical bearing block attached to the compressive testing unit.

The compression load was acted on the specimen with a rate of the range of the ECP specified 0.6 N/mm^2^ per second. The maximum compressive strength (σ) was determined by the division of the peak load (P) by the cross-sectional area (A) of each specimen. Three cubes were examined at 28 days using the following formulae.

1 $$\sigma = \frac{P}{A}$$

### Splitting tensile strength analysis (T)

Twenty-eight days after casting, the measurement of T was carried out following the ASTM C496 standard^24^. The load was adjusted to the ASTM standard at 900 kPa per minute, which is at the midpoint of appropriate load speeds. The test called for a plate strip to be placed on the top and the bottom of each specimen was used as a bearing strip, and the strips were applied for each test. The data collected for these tests included the load as recorded by the testing machine. This data was then recorded to calculate the T of each specimen by dividing twice the peak load (P) by the product of pi, the diameter (D), and the length of the cylinder (L). Three cylinders were examined at 28 days and their average value is reported by following Eq. (2).

2 $$T = \frac{2P}{{\pi LD}}$$

### Flexural strength analysis (R)

The static flexural test was made 28 days after the casting of the specimens. This test was conducted in conjunction with the ASTM C78 standard^25^. For simple beams undergoing third-point loading. The rate of loading for the static flexural test was maintained at 900 kPa per minute. Three beams were examined at 28 days and their average value is reported by Usage of the equation below (3) for the calculation of the rupture modulus which is assessed by the division of the product of peak load (P) and specimen clear span (L) by the product of specimen width (b) and depth (d) squared.

3 $$R = \frac{PL}{{bd^{2}}}$$

### Statistical analysis

Data were statistically analyzed using a two-way analysis of variance (ANOVA test) using SPSS Statistical Package Program version 23. Means of treatments were compared by Duncan multiple range test when the differences were significant^26^. The level of significance in all tests was *P* ≤ 0.05. The results are expressed as means ± standard error (SE).

### Ethical approval

This article does not contain any studies with human participants or animal.

## Results

### Bacterial characterization and identification

It is observed that the synthesis of endospores with bacterial bacilli forms, the positive result for urease test was recorded after 16 h of incubation, and a calcium carbonate powder was observed in NB appended with urea and calcium chloride (NB-U/Ca). A CaCO_3_white powder immediately appeared in the media after the bacterial inoculation and its density reached a high level 7 days after incubation Table 2. The nucleotide sequence via molecular identification was compared with known sequences using the Blastx software (BLAST), The National Knowledge Center for Biotechnology. The bacterial isolate was previously identified in 2019^17^ as *Bacillus cereus* using 16SrRNA gene sequencing and its sequence had an accession number.

**Table 2:** Bio-Characterization of Bacterial Isolate.

Test	
Endospore forming	+ ve
Urease	+ ve
Calcium carbonate precipitation	+ ve
Alkalinity pH	Tolerated till pH 14

### Crack healing

**Figure 1:**
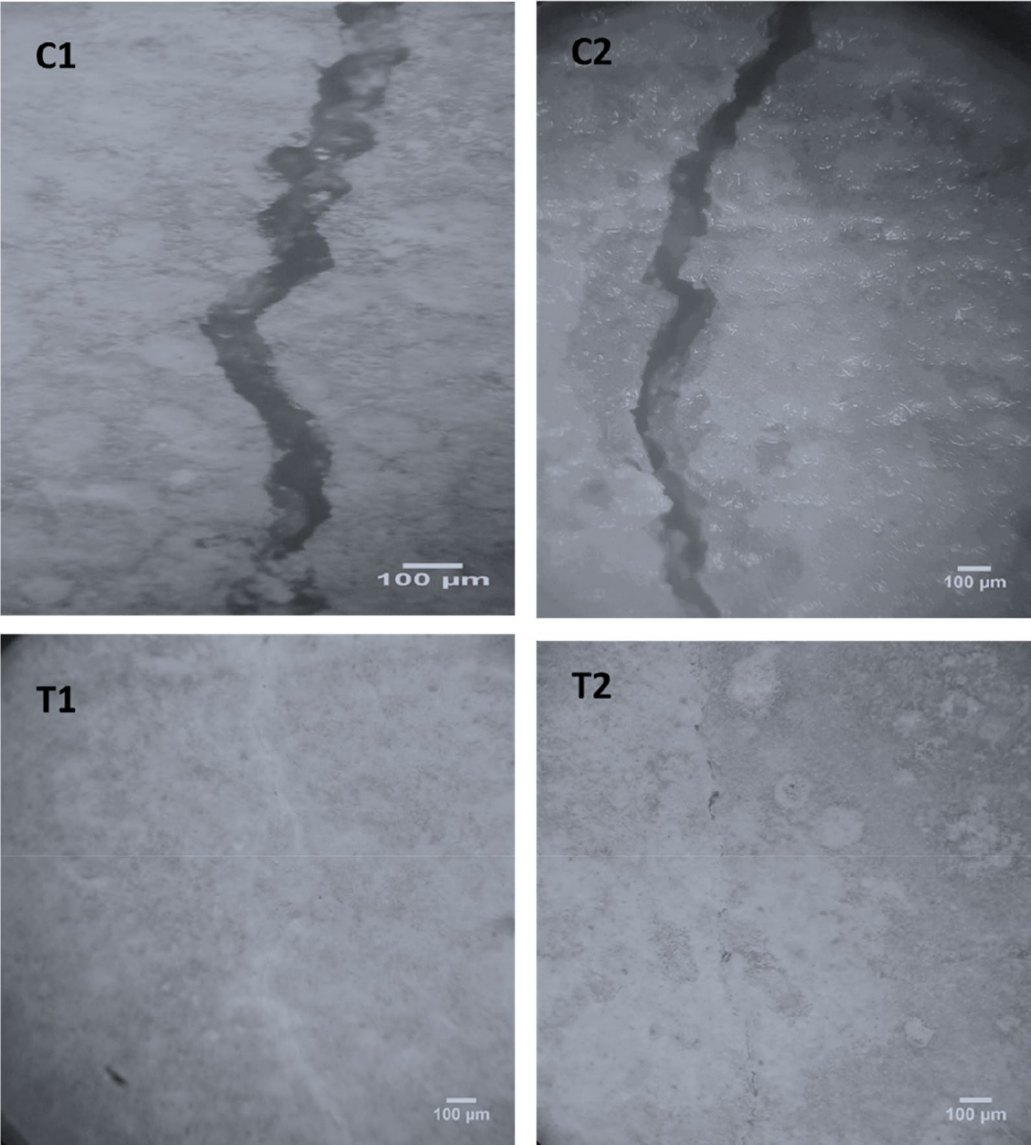
Stereomicroscopic images show crack healing after 7 days pre-cracking.

**Figure 2:**
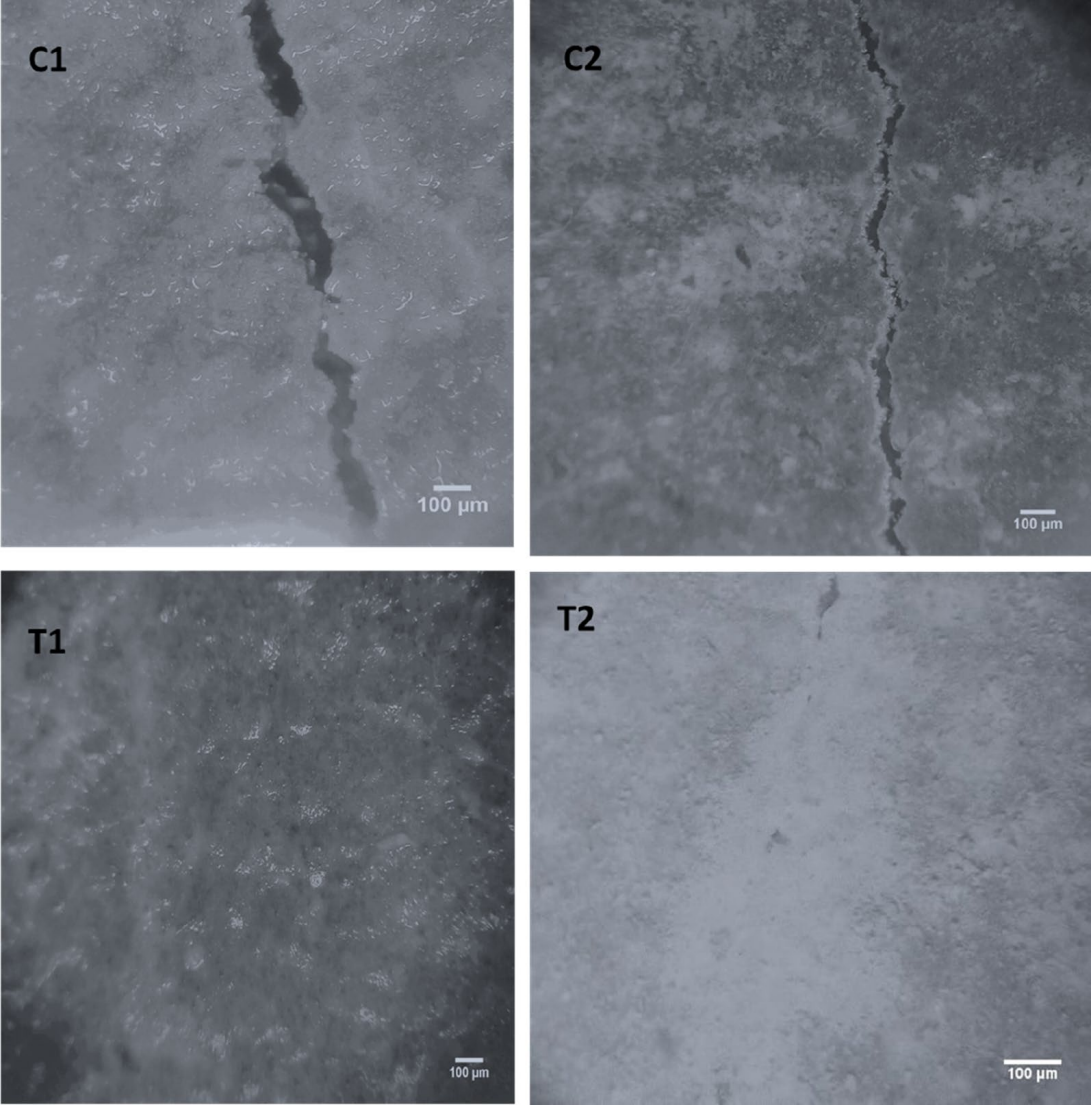
Stereomicroscopic images show the crack healing after 14 days pre-cracking.

**Figure 3:**
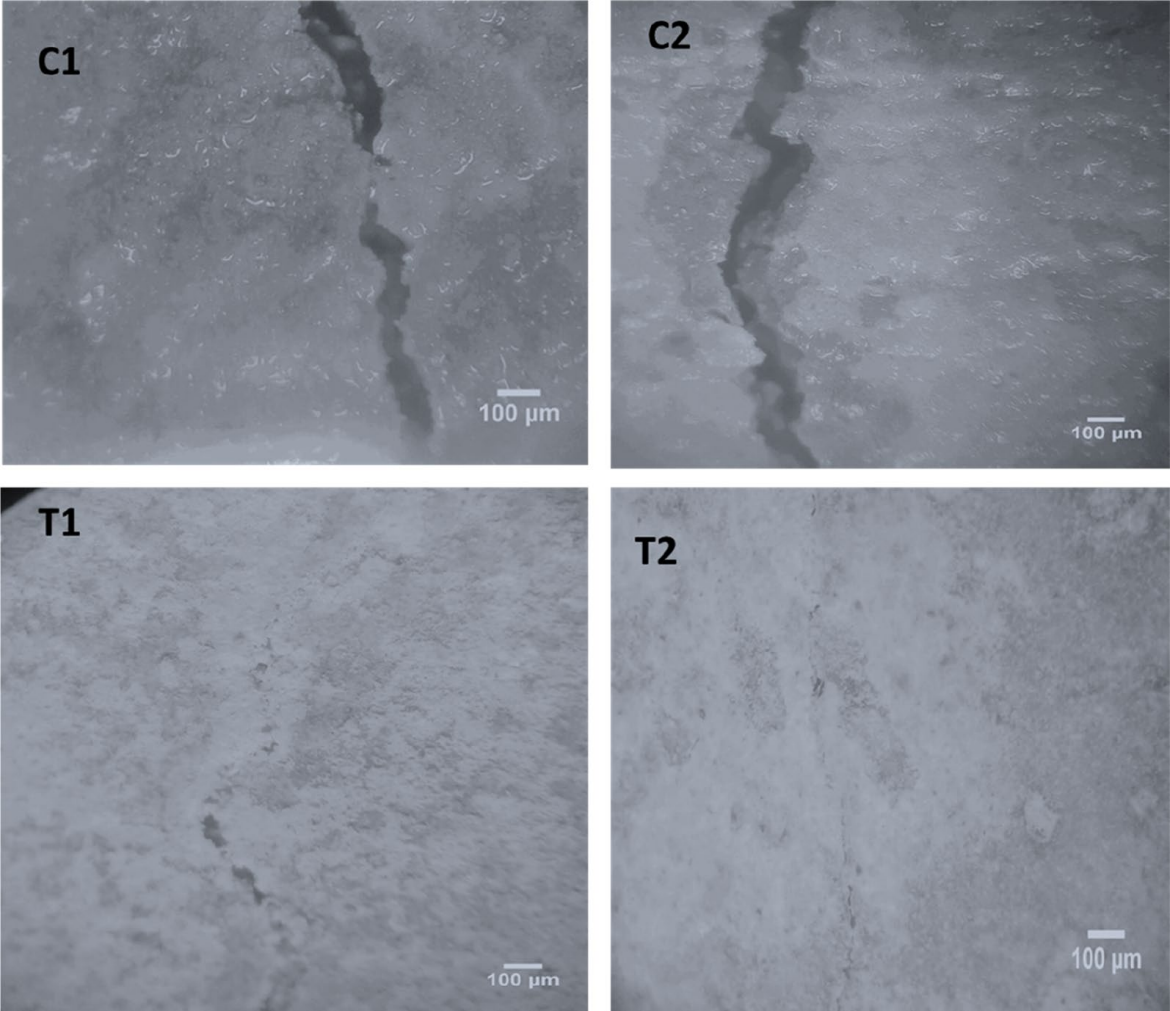
Stereomicroscopic images show the crack healing after 28 days pre-cracking.

The stereomicroscopic images in Figs. 1, 2, 3 show the healing of cracks after 7, 14, and 28 days of previously cracked and water treated mortar specimens. It can be shown that, relative to control samples, all the samples of bacterial spores’ incorporation had positive restoring outcomes. Unlike the direct inoculation technique, it was noted that mixed specimens integrated with wood ash bacterial carrier units displayed the highest healing. Completely healed specimens were observed after 7, 14, and 28 days by shielding the bacteria using wood ash. Direct insertion of bacterial spores, complete healing was only noticed after 7 and 14 days of pre-cracked water treated mortar specimens.

### Mortar Flexural Strength analysis

For the detection of flexural intensity, Samples with bacterial wood ash for the incorporation showed maximum strength and improvements of 22.22, 13.63, and 14.29% in flexural strength at days 7, 14, and 28 compared negative control specimens (Table 3).

**Table 3:** The Flexural Strength of the mortar specimens.

Mortar type	Flexural strength (MPa)		
	7 days	14 days	28 days
C1	1.05 ± 005^f^	1.3 ± 0.13^e^	1.8 ± 0.09^ cd^
C2	1.11 ± 0.09^f^	1.5 ± 0.10^e^	1.9 ± 0.13^bc^
T1	1.05 ± 0.02^f^	1.55 ± 0.12^de^	1.95 ± 0.31^ab^
T2	1.35 ± 0.11^e^	1.65 ± 0.32^ cd^	2.1 ± 0.17^a^

C1: Negative control (all mortar constituents without wood ash or bacterial spores. C2: Positive control (all mortar constituents + wood ash). T1: All mortar constituents + direct bacterial spores inoculation. T2: All mortar constituents + immobilized bacterial spores on wood ash.

^a,b,...^Averages in the table having different subscripts are differ significantly (*P* ≤ 0.05).

### SEM screening

**Figure 4:**
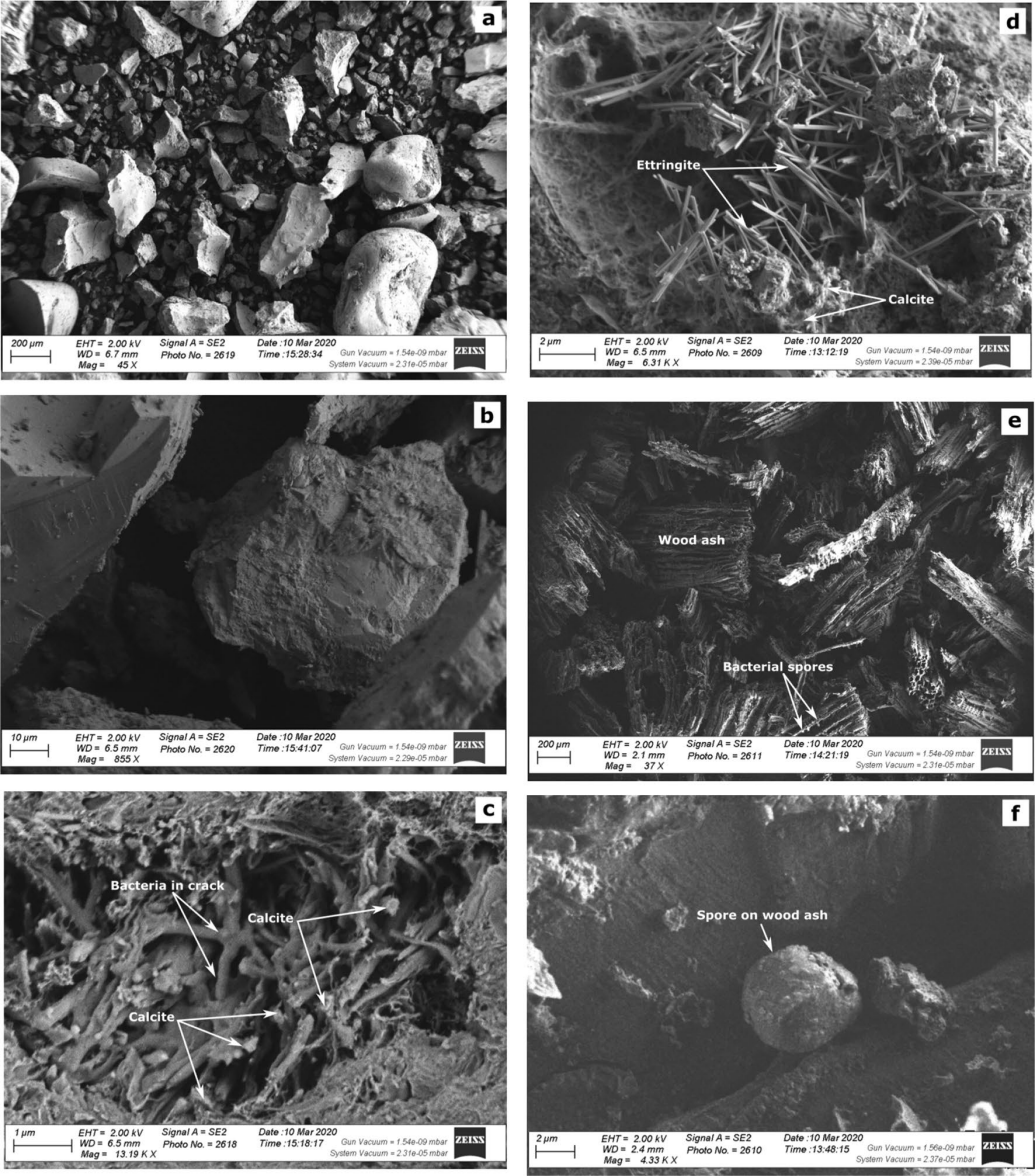
SEM micrographs (**a**) and (**b**) control concrete specimens of loose composition and with no calcite, (**c**) concrete specimen with *Bacillus cereus* MG708176 indicating the presence of calcite crystals and presence of Bacillafilla in cracks, (**d**) shows ettringite and calcite formation, (**e**) shows wood ash saturated with bacterial spores, and (**f**) bacterial spore on wood ash unit.

Calcite and ettringite were observed in bio-concrete cracks as shown in Fig. 4. *BacillaFilla* structure (a combination of calcite, filamentous bacterial cells and Levans glue bacterial secretions) was detected (Fig. 4) in the bio-concrete healed cracks and the concrete frame was again knitted together due to this structure.

### Concrete Slump and Compressive strength analysis

The slump estimation value of the normal concrete was found to be 30 mm in this sample. The slumping value of bio-concrete is recorded to be 40 mm. Table 4 presents the compressive power of self-healing specimens. The recorded data showed that specimens with the bacterial wood ash showed a maximum strength of 41.2 MPa by a 24.7% more than control. The data presented in Table 4 confirmed that bacterial wood ash specimens demonstrated high strength of 4.55 MPa and a tensile strength improvement by 18.9% and a flexural strength improvement by 28.6% more than control.

**Table 4:** The mechanical characteristics of the concrete specimens at 28 days.

Concrete mechanical characteristics	C	T
Compressive strength (MPa)	31 ± 1.90^b^	41.2 ± 3.23^a^
Tensile strength (MPa)	3.69 ± 0.43^b^	4.55 ± 0.30^a^
Flexural strength (MPa)	4.98 ± 0.33 ^b^	6.98 ± 1.06^a^

C: all concrete constituents + wood ash. T: all concrete constituents + wood ash + bacterial spores.

^a,b^Average in the same row having different subscripts are differ significantly (*P* ≤ 0.05).

## Discussion

This study aims to assess the reflection of wood ash units as a bacterial carrier on concrete self-bio-healing efficiency and mechanical properties. Our results revealed that all bacterial integration specimens result in increased bio-healing efficiency more than control and that verified by micro-graphs from the SEM that showed reaction product morphology, which was calcium carbonate and ettringite needle-like phase and is similar to that observed by the cement that was hydrated^9,27,28^. Our findings showed the decreased crack healing of direct inoculation particularly after 28 days may be due to a decline in the feasibility of bacteria survival inside concrete and the loss of bacteria by removal just for the construction of dense microstructures formed^10,11^. Specimens integrated with wood ash carrier units displayed the greatest healing as a consequence of bacterial immobilization and protection from harsh conditions that enable spores persist very long periods^27,29^.

Our study showed that all methods of bacterial integration contribute to increased flexural strength of the mixtures, due to this deposition of CaCO_3_ on the microorganism cell surfaces and inside mortar pores^3^. As cracks occurred, activation of the incorporated bacterial spores, and CaCO_3_ is formed by a supplied nutrients metabolism^30^. This calcium carbonate continuously manufactured by the bacteria, urea, and calcium chloride provided as organic precursors makes the internal structure of concrete more compact.

The rise in compressive strength is in agreement with the results obtained by^3,31–35^ confirm that the bio-healing is a cause of compressive strength improvements in opposition to normal gross aggregates, these improvements may be related to a smaller wood ash units scale. This permitted better packing and the concrete matrix's compaction around them, which gave these specimens much more strength than control specimens^10^. An explanation of the increase in concrete strengths is due to the synergistic effect of wood ash units and calcium carbonate-producing bacteria.

Using wood ash units as a bacterial carrier is a promising material where it is considered a waste that improves the mechanical properties of produced composites leading to better strength and stiffness mixtures^16^ and providing cheap raw material for immobilization process.

## Conclusion

Our results suggested that wood ash works as an efficient immobilization technique to protect bacterial cells from harsh conditions in the concrete. The effective positive role of the immobilized bacteria in the improvement of compressive, tensile, and flexural strengths of concrete was detected by filling the concrete cracks with CaCO_3_ using a calcite-producing bacterium. Our work offers a low-cost raw material for bacterial immobilization as wood ash is regarded a waste and can be used as a potential bacterial carrier that enhances the mechanical characteristics of generated composites.

## Supplementary Information

Supplementary Information.

## Data Availability

All data generated or analyzed during this study are included in this published article.
