# Supplementary material for: Impact of the immobilized Bacillus cereus MG708176 on the characteristics of the bio-based self-healing concrete
Source: Sci Rep. 2023 Jan 10;13:500. doi: 10.1038/s41598-023-27640-1 (PMC9832136; doi:10.1038/s41598-023-27640-1)
Supplement: Supplementary file 1 — Supplementary Information. [file 41598_2023_27640_MOESM1_ESM.docx]

**Impact of the immobilized *Bacillus cereus* MG708176 on the characteristics of the bio-based self-healing concrete**

Amany M. Reyad^a*^ Gehad Mokhtar ^b^

1. Lecturer of Microbiology, Department of Botany, Faculty of Science, Fayoum University, Egypt.

b) Lecturer of Civil Engineering, Future high Institute of Engineering in Fayoum, Egypt.

* Correspondence should be addressed to Amany M. Reyad; [amr01@fayoum.edu.eg](mailto:amr01@fayoum.edu.eg)

cps/eV

30


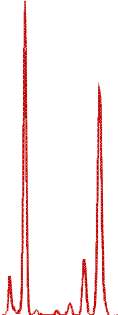

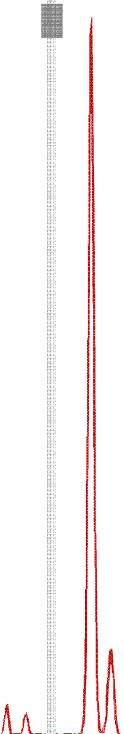

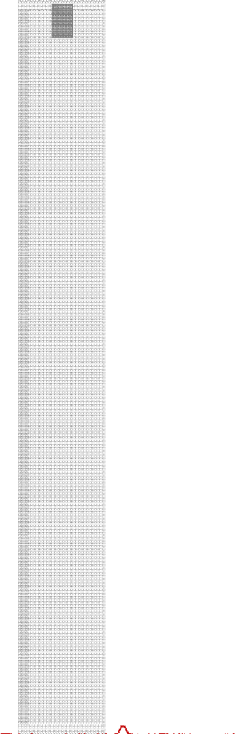

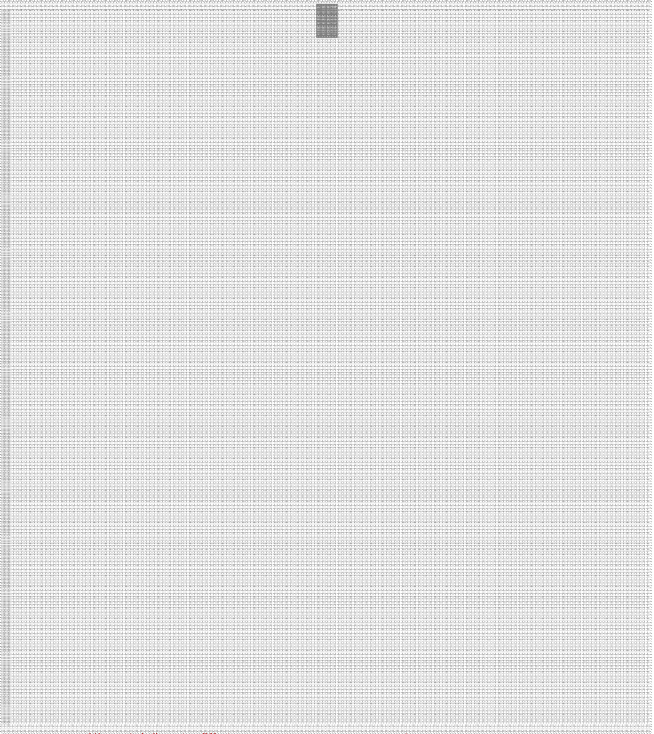

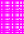

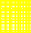

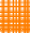

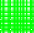

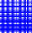

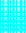

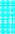

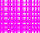

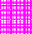

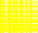

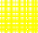

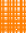

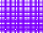


1

2

3

Fe Ca

S F N

O Si

C Na Al S

Ca

Fe

25

20

15

10

5

0

2 4 6

8 10 12

14 16

18 20

Energy [keV]

Fig s1. EDX shows element composition of wood ash

Table s1. Element percentage in wood ash sample

| Element | Atom Number. | Mass [%] | Atom [%] |
| --- | --- | --- | --- |
| Carbon | 6 | 5.99 | 9.26 |
| Oxygen | 8 | 55.42 | 64.29 |
| Nitrogen | 7 | 2.21 | 2. 92 |
| Aluminum | 13 | 1.74 | 1.19 |
| Silicon | 14 | 5.53 | 3.65 |
| Sulfur | 16 | 0.92 | 0.53 |
| Calcium | 20 | 37.48 | 17.36 |
| Iron | 26 | 1.42 | 0.47 |
| Fluorine | 9 | 0.00 | 0.00 |
| Sodium | 11 | 0.38 | 0.31 |
